# Improving Upper Extremity Function and Quality of Life with a Tongue Driven Exoskeleton: A Pilot Study Quantifying Stroke Rehabilitation

**DOI:** 10.1155/2017/3603860

**Published:** 2017-12-18

**Authors:** Stephen N. Housley, David Wu, Kimberly Richards, Samir Belagaje, Maysam Ghovanloo, Andrew J. Butler

**Affiliations:** ^1^Department of Physical Therapy, Georgia State University, Atlanta, GA, USA; ^2^School of Applied Physiology, Georgia Institute of Technology, Atlanta, GA, USA; ^3^School of Nursing & Health Professions, Georgia State University, Atlanta, GA, USA; ^4^Department of Veteran's Affairs, Atlanta Rehabilitation Research and Development Center of Excellence, Decatur, GA, USA; ^5^School of Medicine, Emory University, Atlanta, GA, USA; ^6^School of Electrical and Computer Engineering, Georgia Institute of Technology, Atlanta, GA, USA; ^7^Neuroscience Institute, Joint Center for Advanced Brain Imaging, Center for Behavioral Neuroscience, Georgia State University, Atlanta, GA, USA

## Abstract

Stroke is a leading cause of long-term disability around the world. Many survivors experience upper extremity (UE) impairment with few rehabilitation opportunities, secondary to a lack of voluntary muscle control. We developed a novel rehabilitation paradigm (TDS-HM) that uses a Tongue Drive System (TDS) to control a UE robotic device (Hand Mentor: HM) while engaging with an interactive user interface. In this study, six stroke survivors with moderate to severe UE impairment completed 15 two-hour sessions of TDS-HM training over five weeks. Participants were instructed to move their paretic arm, with synchronized tongue commands to track a target waveform while using visual feedback to make accurate movements. Following TDS-HM training, significant improvements in tracking performance translated into improvements in the UE portion of the Fugl-Meyer Motor Assessment, range of motion, and all subscores for the Stroke Impact Scale. Regression modeling found daily training time to be a significant predictor of decreases in tracking error, indicating the presence of a potential dose-response relationship. The results of this pilot study indicate that the TDS-HM system can elicit significant improvements in moderate to severely impaired stroke survivors. This pilot study gives preliminary insight into the volume of treatment time required to improve outcomes.

## 1. Background

Stroke is one of the leading causes of long-term disability [1] with 795,000 individuals * *experiencing a stroke in the United States annually [2]. Of those who survive, 80% experience significant upper extremity (UE) motor impairment [3] requiring long-term rehabilitation to regain movement in the * *impaired extremities [4]. Successful rehabilitation techniques involve intensive, repetitive practice that actively engages the participant in goal-oriented and task-specific activities.

One such method involves the use of robotic devices to assist with delivering an optimal dose of * *therapy [5]. Recent advances in rehabilitation techniques, including robotic therapy (RT), have greatly * *increased the level of function patients can achieve. Several studies have observed equivalent outcomes * *with either robotic therapy or one-on-one rehabilitation [6–9]. The results of these studies indicate that * *RT provides reliable, reproducible treatment while providing objective measures of kinematic performance [10]. These techniques cause changes in the brain's neural pathways, allowing patients to * *regain function through altered and adapted networks [11]. Despite these developments, prognosis * *remains poor and treatment interventions are limited for chronic stroke survivors who possess limited active range of motion [12].

Many robot-assisted technologies require participants to actively initiate hand motion before the robot can assist completion of the movement task. This requirement limits its applicability to the most impaired stroke survivors. Assistive technologies, used to control and modify a stroke survivor's environment, are commonly utilized [13] with clinically significant outcomes [14] by even the most impaired stroke survivor. However, RT and assistive technologies are rarely hybridized to take advantage of the potential additive benefits.

One such RT device is the Hand Mentor™ (HM). The HM device was designed for individuals with residual UE impairments. The goal of using the device is to improve active range of motion (AROM) and strength in the distal musculature of the paretic limb of patients with poststroke weakness. To improve applicability of RT to stroke survivors, the robotic device used in the current study was interfaced with a known assistive technology that utilizes a wireless headset to detect the movements of a small magnetic tracer temporarily adhered to the tongue (Tongue Drive System: TDS) [15]. The hybrid TDS-HM system encourages users to actively move their paretic hand while using synchronous tongue commands to drive motion of the robotic device. However, active motion of the wrist and hand is not necessary to drive the unit. The TDS-HM device provides new experiences and learning that incorporate cognitive planning, timing, and increasing difficulty through computer-game-like training programs for motor learning and control. Within this motor learning framework, skill acquisition can be understood as practice-dependent reduction of kinematic and dynamic performance errors detected through the participant's visual and proprioceptive sensory channels.

Consistent with principle of motor learning [16], that training must continually challenge learners, the device increases difficulty levels based on the performance. The TDS-HM enabled individuals with little or no active hand movement to participate in a mode of therapy that has the potential to remodel the brain's neural pathways [17].

The theoretical construct that underpins the notion that the TDS-HM will enhance structural and functional recovery relies on interventions being designed to modulate neural activity in sensorimotor regions to facilitate activity-dependent neuroplastic changes in the brain [18]. With chronic activity modulation, short-term, activity-dependent in brain signaling can begin to be transferred to the propagation of new pathways and possibly circumvent existing damaged tissues [19–24]. Furthermore, arm muscles are preferentially unilaterally innervated, whereas tongue muscles are bilaterally innervated. This is important for our work because even in severe strokes in which arm function is severely impaired, tongue muscles remain intact (with the exception of caudal brain stem lesions).

Extensive cortical representation overlap has been identified in the motor cortices of the hand and tongue [25]. These two regions are highly interconnected functionally, taking part in synchronous activation during independent hand and tongue movements [26, 27]. Further regional interconnectivity * *occurs in individuals suffering from phantom limb pain, who show extensive cortical activity in deafferented hand representations when purposeful lip and mouth movements are made [28]. These data * *also suggest extensive functional reorganization occurs from the lip and mouth region to the hand cortical * *region following deafferentation. Other data purport that topographical alterations of the sensorimotor * *cortex can shift the motor representation of the tongue into the region of the hand [29].

Motor tasks act as drivers for neuroplastic change. For the tongue, novel nonverbal motor task * *training has been shown to drive neuroplastic changes in human and nonhuman models, with alterations * *being observed up to 24 hours after an intervention [30, 31]. Boudreau et al. hypothesized that these short-term changes seen in humans may initiate and propagate the long-term neuroplastic changes * *required for structural reorganization of neural circuitry [31].

In this pilot study we aim to improve upper extremity function and quality of life in people with * *moderate to severe impairment with a tongue driven robotic exoskeleton. We anticipate that allowing stroke survivors with UE impairments to synchronously use their tongue to control a UE robotic device will empower them to play a more active role in their therapy encouraging learning that incorporates cognitive planning, timing, and increasing difficulty. We hypothesize 30 hours of TDS-HM training will * *improve motor performance tracking kinematics, reduce UE motor impairment, and improve quality of life. Additionally, we will investigate the dose-response relationship between daily training and motor performance tracking.

## 2. Methods

### 2.1. Participants

Volunteers between the ages of 18 and 85 with a moderate to severe unilateral ischemic or hemorrhagic stroke within the previous 3–36 months were recruited. Moderate stroke is defined as 21–50 and severe stroke is defined as 0–20 on the upper extremity portion of the Fugl-Meyer Motor Assessment (FMA/UE) [32, 33]. Inclusion criteria included persistent hemiparesis as indicated by a score of 1–3 on the motor arm item of the NIH Stroke Scale [34] and significant impairment that limited their activities of daily living (ADL). Those with clinically significant comprised mental status within three days of enrollment, severe receptive or expressive aphasia, any weakness or diminished sensation of the tongue as determined by standard cranial nerve clinical testing, hemispatial neglect, or score of >2 on the Modified Ashworth Scale were excluded. Additionally, due to the physical nature of the robotic rehabilitation, those with significant flexion contracture at any joint of the UE that would not allow safe and proper set-up on the Hand Mentor device, as indicated by a score of >2 on the Modified Ashworth Scale [35], were excluded.

Prior to the intervention, all participants were informed about the study and any related potential risks. Participants then signed informed consent approved by the institutional review board of Georgia State University (Ref. H13510). A total of six stroke survivors (60.8 ± 11.6 years old (mean ± SD)) with UE hemiparesis resulting from unilateral stroke (21 ± 9.26 months after stroke (mean ± SD)) met inclusion criteria and were enrolled in this pilot study. Stroke survivors were not enrolled in formal rehabilitation activities while participating in this study; however, participants were encouraged to maintain their normal activities.

### 2.2. The Hand Mentor

The Hand Mentor (HM) device was designed for use by individuals with UE impairments after stroke. The goal of using the device is to improve range of motion (ROM) and strength in the distal musculature of the paretic limb of patients with hemiparesis and weakness through highly intensive, task-specific, and interactive practice [36, 37]. Results from previous clinical trials demonstrate that use of the HM improves self-reported functional hand use and health related quality of life measures [9, 38, 39], while a recent home-based telerehabilitation intervention found that the use of the HM results in statistically and clinically significant improvements in UE clinical outcome measures [40]. Further, a recent large-scale, RCT found the HM to be successful at producing significant improvements in UE motor outcomes for subacute stroke survivors at a similar level to traditional rehabilitation [41]. The HM is unique in design as it provides targeted RAT for the wrist, where most other robotic interventions are initiated proximally.

### 2.3. The Tongue Drive System

Tongue Drive System (TDS) is a wireless assistive technology that was developed to allow patients with high level spinal cord injury to drive a power wheelchair [42]. The TDS utilizes the voluntary movements of the tongue to enable users to control their environments, completely independently of the ability to speak [15, 43], by only requiring users to be able to move their tongue to user-defined positions repeatedly and consistently [44]. The TDS utilizes a wireless headset with a pair of extensions that position magnetic sensors on each side of the face which are used to track a small magnetic tracer (5 mm in diameter, 1.1 mm thick) that is temporarily glued near the tip of the user's tongue by oral adhesive (PeriAcryl, GluStitch Inc., Point Roberts, WA). Changes in tongue position inside the oral cavity result in changes in the magnetic field. This information can be tethered to commands that are sent wirelessly to a PC to access computers or other assistive technologies.

### 2.4. Hybrid TDS-HM Intervention

The hybrid TDS-HM system interfaced the wearable TDS with the HM, so that three discrete commands (up and to the right, down and to the left, and neutral) given by movements of the tongue provide assistance with wrist extension and flexion in the robotic device. Taken together, the TDS-HM system allows users to use active wrist movement in combination with tongue-derived assistance to track goal-oriented target waveforms presented on the computer screen. Tracking performance was quantitatively assessed as the magnitude of difference between goal and observed location during the various training paradigms. This concept is further described in Section 2.7. The training selected required the participants to navigate sine, triangle, and random rectangle waveforms through synchronous wrist motion and tongue commands. The participants were instructed to continually attempt active wrist motion in the robotic device while simultaneously issuing the corresponding command with the tongue to track the waveforms. However, active wrist or hand movements were not required. Participants with little or no active control are able to accurately track the waveform entirely by the TDS commands.

### 2.5. Training Protocol/Experimental Design

**Figure 1:**
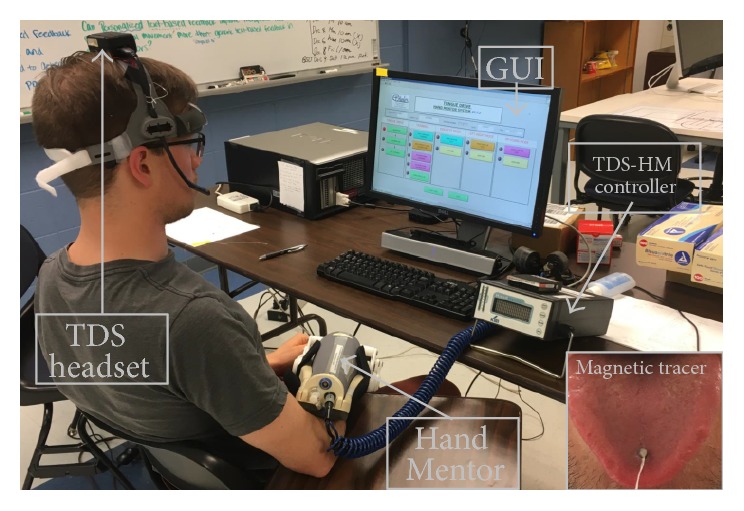
Experimental set-up. Top center, the graphical user interface (GUI) that participants view during game training. Bottom center, the Hand Mentor (HM) device worn on the right (paretic) upper extremity. Left, healthy volunteer with TDS headset affixed to the head. Magnetic sensors are positioned bilaterally to capture the maximal oral area. Bottom right, the small magnetic tracer used to control the HM device. Bottom center, the HM controller with red safety button to be used to immediately stop the training.

The intervention was controlled for frequency (2 hours, 3 times per week) and duration (5 weeks). Participants were asked to complete a total of 15 two-hour sessions of RT with the TDS-HM. The training intensity was set at three times a week with all 15 sessions to be completed within five consecutive weeks. This training paradigm was chosen to allow participants to aggregate a total of 30 hours of therapy, a threshold at which objective functional improvements generally occur [45]. Prior to the first training session, a preliminary acclimatization period was allowed to ensure that participants understood the exercises and device set-up (Figure 1). All sessions were supervised by a licensed physical therapist (KR) trained in the use of the TDS-HM.

During training sessions, participants sat in an upright position in front of a 22′′ computer monitor. Participants wore the external TDS headset, and a small magnetic tracer was attached to the participant's tongue using oral adhesive PeriAcryl (GluStitch Inc., Point Roberts, WA). The HM was then put on the participant's paretic arm and wrist with elbow positioned at 90° flexion and forearm on an armrest. Participant's active and passive wrist range of motion were measured goniometrically [46] to calibrate the sensitivity (gain) of the HM device. No support was provided at the level of the proximal arm, so that participants could position and move their upper arm freely. Possible compensatory trunk movement or abnormal wrist movements were monitored and manually prevented by the physical therapist supervising the therapy.

**Figure 2:**
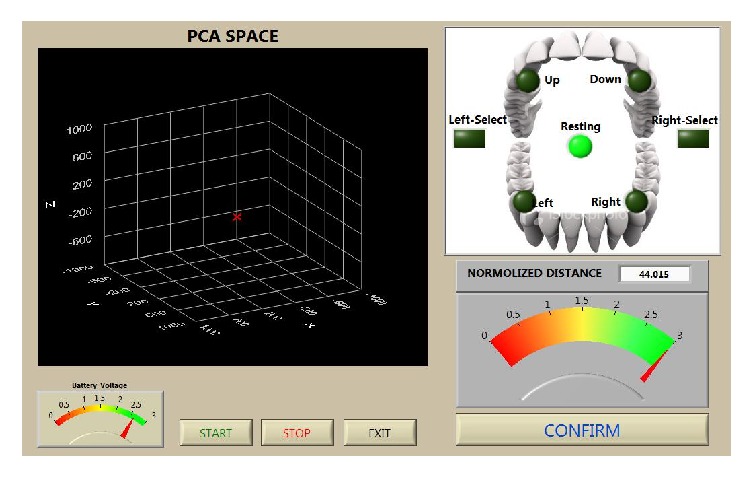
Pretraining calibration. Pretraining graphical user interface (GUI) showing the virtual 3D representation of the magnetic tracer, attached to the user's tongue, in the oral space. Principal component analysis (PCA) space comprised transformed vectors, identifying unique tongue positions in 3D space.

During each of the 15 training sessions, a series of calibrations were completed to ensure the accuracy of the commands issued by the TDS system. Although a detailed description of the TDS calibration has been previously described [42], a brief summary of the process requires the participant, prior to training, to interact with the graphical user interface (GUI) to define three discrete and reproducible command locations (up and right, down and left, and neutral) in three-second intervals (Figure 2). The calibration uses principal component analysis (PCA) to extract relevant features of each of the three defined commands. These commands are repeated 10 times for each position, while 12 variable vectors are extracted and used to calculate eigenvectors and eigenvalues of the three-dimensional intraoral space. The three eigenvectors with the largest eigenvalues were then selected to set up a feature matrix that was used to determine when the participant was issuing a command with the tongue. This process typically takes 10 minutes to complete. As participants become more comfortable with the calibration process calibration time was reduced.

**Figure 3:**
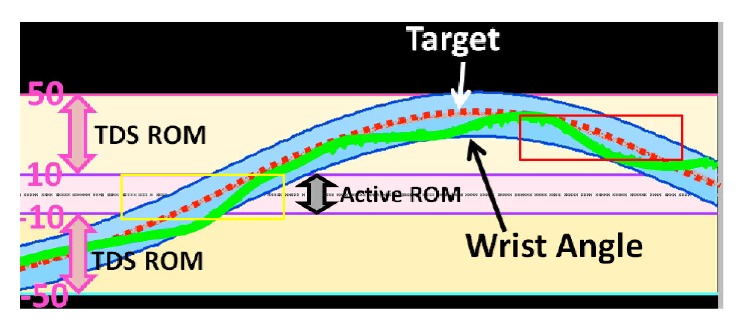
Example of visual feedback given to participants. Active ROM (pink zone) represents the magnitude of motion in which the participant is able to volitionally move her or his wrist. The Tongue Drive System (TDS) ROM, where tongue control can command the HM, is the portion of available ROM in which the stroke survivor is not able to voluntarily move. Vertical pink errors represent the TDS ROM above and below the stroke survivors active ROM. Highlighted yellow and red regions represent portions of tracking waveforms in which the stroke survivor will exclusively use the hand and the tongue and hand, respectively.

**Figure 4:**
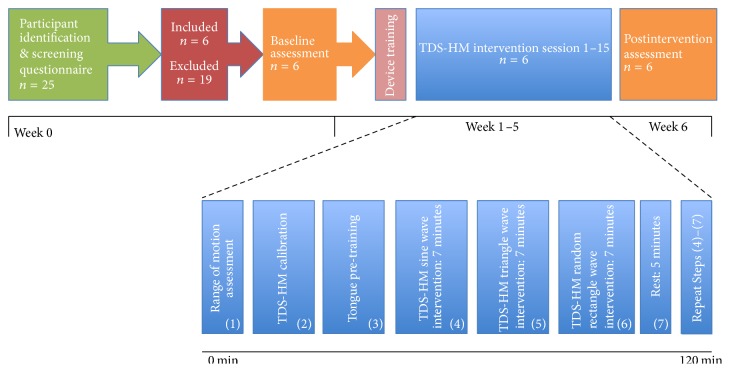
Participant flow through the study. Participant identification based on inclusion and exclusion criteria, followed by phone screening by research clinician. Baseline assessment and device training were completed upon first visit to the research setting. During each of the 15 training sessions, participants completed three sets, consisting of approximately six to eleven trials of sine, triangle, and random rectangle waveforms (40 seconds), in order, lasting four–seven minutes each. A short rest period of up to five minutes was allowed between each set to prevent muscle fatigue.

Following successful calibration, participants were then instructed to move their paretic UE, with synchronized tongue commands, in order to replicate the goal movement pattern displayed on the monitor. The target and observed movements were explained to the participants. The participants were instructed that during the training, they were to track the target and use wrist angle as feedback (Figure 3) to make their movements as accurate as possible (i.e., lowest root mean squared error (RMSE)), duplicating the goal movement pattern. Participants completed three sets, consisting of approximately six to eleven trials of sine, triangle, and random rectangle waveforms (40 seconds), in order, lasting four–seven minutes each. A short rest period of up to five minutes was allowed between each set to prevent muscle fatigue (Figure 4). Following each session, the TDS headset and HM robotic devices were removed. The supervising therapist then assisted with removal of the magnetic tracer.

### 2.6. Clinical Outcome Measures

Preassessments of all outcome measures for all participants were completed within one week prior to training session one, and postassessments were completed up to one week after the last training session. Outcome assessment was deferred on training days to reduce the confounding effects of fatigue.

Active (AROM) and passive (PROM) range of motion for wrist extension were measured goniometrically, as standardized by Norkin and White [46]. Measurements of AROM and PROM for wrist flexion and extension were assessed prior to each daily training session to calibrate the HM sensitivity (gain). To account for the device weight and the physical interaction between the arm and the device, PROM and AROM measures were completed when the participants had the HM donned.

The Wolf Motor Function Test (WMFT) determines the time required to perform 15 everyday tasks with each UE. The functional items range in level of difficulty, requiring first single and proximal joint motions and progress to combined joint motions involving the distal extremity. The WMFT has been validated for use with acute to chronic stroke survivors [47–49].

The FMA/UE Scale is a 33-item test with each item scored on a 3-point ordinal scale that measures motor function and recovery after stroke [32]. Scores range from 0 to 66 (normal function) [50]. The FMA/UE is a reliable and valid tool for measuring UE impairment following stroke [51].

The Stroke Impact Scale (SIS) is a full spectrum health status inventory. It is a stroke-specific, self-report measure composed of 59 items, distributed in eight separate domains examining strength, hand function, mobility, activities of daily living, emotion, memory, communication, and social participation [52] as well as the newly hypothesize physical cluster [53].

### 2.7. Robotic Outcome Measures

Kinematic data collected by the HM was used to evaluate motor performances. To evaluate hand and wrist motor control during the training sessions, the RMSE was used to determine how closely the participants followed the target by calculating the difference between target and observed tracking performance. A consistent decrease in RMSE over time indicates more accurate tracking and motor learning [54, 55]. TDS-HM usage is reported in terms of total and daily usage time (minutes) of the device during the five-week period. The overall RMSE for a given session's block was calculated using the following formula:

(1) $$\begin{array}{c} {RMSE}_{overall}=\sqrt{\frac{\sum_{i=1}^{n}{\left({\hat{y}}_{i}-y_{i}\right)}^{2}}{n}}. \end{array}$$

### 2.8. Data Analysis

Observed and target tracking performances were recorded (50 Hz sampling rate), during each therapy session, and transferred to a customized Microsoft Access database. Waveform tracking error, as assessed by RMSE, was calculated for the first sine waveform of each therapy session, by comparing the observed tracking to the target tracking, where *y*_*i*_ is the observed tracking and ${\hat{y}}_{i}$ is the target tracking. RMSE calculations were completed by sampling approximately the same length block (first 5 minutes) of every trial. The sampling method was chosen to reduce the impact of fatigue on performance following repeated training bouts [56] and attenuate the occurrence of experience-dependent plasticity within the cortical-cerebellar and cortical-striatal neural systems during the fast learning phase [57]. The first 40 seconds of each block were discarded to account for initial pneumatic pump filling and allow the participant to reach a steady-state tracking performance. The remaining time in the block was used for analysis.

### 2.9. Statistical Analysis

Data were checked for accuracy against data entry forms and expressed as means, medians, SDs, and ranges calculated using Microsoft Excel. Total time usage for each waveform was extracted from the Microsoft Access database and calculated in Excel. All remaining analyses were completed using SPSS, version 22 (IBM, Armonk, NY). Changes from baseline in impaired hand FMA/UE, WMFT, and SIS domain scores were compared to the corresponding estimated values for the minimum clinically important difference (MCID) in chronic stroke for these measures [58–62]. Changes in functional outcome scores from baseline were analyzed using paired* t*-tests. Linear mixed-effect modeling was used to assess the effect of time on changes in tracking performance (RMSE) from baseline. Time × RMSE was the main interaction of interest. A linear mixed-effect model was chosen in order to accommodate repeated measures designs while not making assumptions of independence among all data points. An autoregression order 1 (AR(1)) structure was chosen because it does not specify that the covariance between observations on the same participant must be equal, but may increase with lag [63]. These approaches were chosen because they are better able to accommodate missing data points from cases in which individual participants did not complete all fifteen training sessions or the postassessment. A linear regression model was utilized to determine the strength of association between training time (minutes per day) and RMSE tracking performance. The level of significance was set equal to 0.05 and all tests were 2-tailed.

### 2.10. Sample Size Calculation and Estimation of Effect Size

Due to the nature of the pilot study, a prospective sample size calculation was not conducted. Estimations of effect size were used to determine the effect TDS-HM had on improving motor performance tracking, UE functioning, and UE impairment between pre- and postintervention. Data were entered into the effect size calculator G*∗*Power (version 3.1.9.2) [64]. Effect sizes were specified as Cohen's *d* = |*μ*_1_ − *μ*_2_|/*σ*, where pre- and postmeans are defined as *μ*_1_ and *μ*_2_, respectively, and the pooled standard deviation as *σ* [65]. Effect sizes were then reentered into G*∗*Power to complete an a posteriori power analysis to provide sample size estimates for future studies [64].

## 3. Results

Figure 4 shows the flow of the participants through each stage of the study. Six stroke survivors (60.8 ± 11.6 years old (mean ± SD)) with UE hemiparesis resulting from unilateral middle cerebral artery (MCA) territory infarcts (21 ± 9.26 months after stroke (mean ± SD)) met inclusion criteria and were enrolled in this pilot study. All six participants showed moderate to severe UE impairment (25.7 ± 14.9) on the FMA/UE [32, 33] secondary to corticospinal tract infarcts. No brainstem or multiple vascular territory infarcts were present. Overall the TDS-HM training was safe and well tolerated. No adverse events occurred. Baseline characteristics including demographic information, comorbid conditions, lesion characteristics, and disclosed medications are presented in Table 1.

Five stroke survivor participants completed the pilot study, consisting of 15 training days of TDS-HM training over 5 weeks. One participant dropped out after 10 days of TDS-HM training due to scheduling conflicts and lack of motivation. However, this participant completed all postintervention outcome measure assessments so his/her data was included in all analyses. Means and SD for clinical outcome measures for participants at baseline and postintervention are presented in Table 2.

### 3.1. Clinical Outcome Measures

All clinical assessments at postintervention showed improvement over the course of the study (Table 2). At the end of the TDS-HM intervention (week 6), participants improved 5.5 points (21.43%) on average on the FMA/UE scale (*p* = 0.05) achieving the previously validated MCID between 4.25 and 7.25 points [62], indicating clinically significant improvements. On average, participants showed statistically significant improvements in wrist PROM (+30.52%, *p* = 0.034) and moderate, nonsignificant improvements (+56.15%, *p* = 0.088) in wrist AROM at postintervention assessment. Modest, nonsignificant (+3.02%, *p* = 0.801) improvements were observed in mean WMFT performance times, with one participant being unable to perform any WMFT task at baseline or postintervention assessment (data not shown). Participant's self-reported quality of life measures showed improvements across all domains of the SIS, with clinically (+11.46 points) and statistically significant (*p* = 0.028) improvements noted for the strength domain.

### 3.2. Robotic Outcome Measures

Summaries of the robotic outcome measures are presented in Table 3. Throughout the 15-day intervention, two severely impaired participants (P5 and P6, baseline FMA/UE scores of 4 and 20, resp.) [33] were unable to complete the prescribed daily dose of training which we defined as three sets. Those participants were only able to complete one set of training waveforms on the majority of the training days (>10 days) and often (>6 days) could only complete one set, the sine waveform training. Additionally, these two participants did not consistently participate in the remaining triangle and rectangle waveform training. As a result, total device usage time was lower than expected for these two people. To control for differences in total device usage and limited exposure to different waveforms, the sine waveform was chosen for all robotic tracking error outcome measure analyses. The sine wave was the initial waveform participants completed each intervention session, and every participant was exposed to at least one sine waveform each training day, thus attenuating the occurrence of experience-dependent plasticity within the cortical-cerebellar and cortical-striatal neural systems during the fast learning phase [57].

Total (371.86 ± 228.79 min) and daily (24.79 ± 16.39 min) mean device usage are reported for all waveforms completed to account for total training exposure. To explore the interaction of dosage time and performance (RMSE), further independent analysis of the sine waveform was required. The mean sine wave training time (133.84 ± 67.85 min) had large variability among the participants, ranging from 46.95 to 233.14 minutes. Daily sine waveform training also varied greatly between participants (3.19 to 24.98 min).

**Figure 5:**
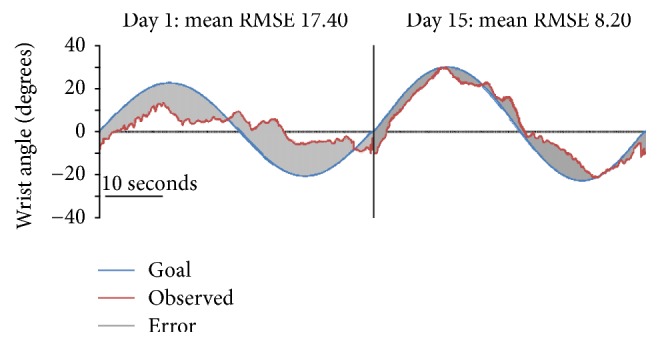
Mean tracking performance. Mean tracking performance from the first training session to the last training session. The solid blue line indicates the target sine waveform. The solid red line indicates the observed tracking. The grey shaded region indicates the tracking error.

Preliminary pre/postanalysis of changes in tracking performance revealed significant moderate decreases (52.87%, *p* = 0.007) in tracking error (RMSE) across all participants, indicating improved tracking performance with the TDS-HM intervention (Figure 5). The first and second tracings in Figure 5 present the mean tracking performance for a single 40-second sine waveform during the initial and final TDS-HM training sessions for all subjects. For both tracings, target and observed tracking performances are represented by blue and red lines, respectively, with the error $\left({\hat{y}}_{i}-y_{i}\right)$ represented by the shaded grey area. Decreases in tracking error can be clearly observed between the two tracings indicating that improvements in participants' ability to accurately track the target waveform had occurred during 15 TDS-HM training sessions.

**Figure 6:**
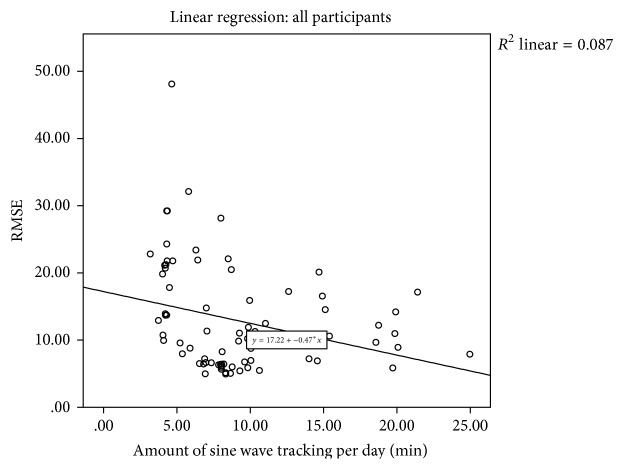
Preliminary dose-response relationship between RMSE and daily sine wave practice time. Linear regression models exploring preliminary dose-response relationship between RMSE and daily sine wave practice time for all participants.

Further repeated measures analysis, using linear mixed-effect modeling, found no significant effect from intervention time on RMSE tracking performance, *f*(14,59) = 1.246, *p* = 0.268. Linear regression modeling, examining the relationship between training time and tracking performance across all participants, found increasing training time to be a significant predictor of decreases in RMSE tracking error (*β* = −0.473, = 0.006) (Figure 6(a)).

## 4. Discussion

This preliminary study aimed to evaluate the effects of combining a robotic-assisted rehabilitation device (HM) with an assistive technology called the Tongue Drive System to improve function and quality of life for stroke survivor. The TDS and HM systems were chosen in order to synchronously activate tongue and hand motor areas in the cortex. We hypothesized 30 hours of TDS-HM training will improve motor performance tracking kinematics that transfer to reduced UE impairment.

The study demonstrated that moderately to severely affected stroke survivors (as evidenced by low mean enrollment FMA/UE scores) can safely and feasibly participate in active, prolonged, repetitive task practice using TDS. UE motor impairment decreased during the treatment period, as evidenced by significant improvements in FMA/UE scores and PROM. Moderate, nonsignificant improvements in AROM were observed. On average, participants were able to complete the WMFT test in less time and were able to successfully complete more tasks after completion of TDS-HM training; however, these changes did not represent clinical or statistically significant improvements. Additionally, these preliminary data suggest that the TDS-HM intervention has the potential to elicit improvements in quality of life measures across all physical dimensions of the SIS for both moderate and severely impaired stroke survivors. All participants experienced decreases in tracking error, suggesting that improvements in motor performance occurred after training with the TDS-HM. Although, when accounting for the repeated measures design, nonsignificant improvements in tracking error occurred, we believe improvements in waveform tracking transferred to clinically significant improvements in UE function in stroke survivor participants. In agreement with previous meta-analytic work by Lohse et al. [5] investigating dose-response relationship between treatment time and motor improvement, our regression modeling indicates that longer daily treatment time is a significant predictor of motor performance improvement for the majority of subjects.

Although previous studies have supported FMA/UE and WMFT scores construct validity for the involved UE [49, 66], our results indicate varied changes at postintervention assessment between the two measures. Mean improvement in FMA/UE score following the TDS-HM intervention (5.5) was similar to the change seen with previous RT studies, 2.5 to 5.3 [67–70], while reaching clinical and statistical significance, whereas nonsignificant (*p* = 0.801) changes in WMFT mean performance times did not achieve clinical significance seen in previous RT studies [41, 60]. The discrepancy may in part be due to the heterogeneous abilities in the participants. The WMFT may not have been sensitive to our severely impaired stroke survivors because of a floor effect of task difficulty [66, 71]. Exploratory analysis removing the two most severely impaired participants reveals an overall improvement in WMFT performance time (3% to 10%), presenting a greater positive trend toward the MCID of 19%. As an alternative to the WMFT, future studies may utilize the robotic kinematic data to monitor changes in the magnitude and peak wrist range of motion. Monitoring this data throughout the course of subsequent TDS-HM interventions may provide more sensitive measures capable of detecting changes in severely impaired stroke survivors. It must be noted that although the HM is capable of detecting changes in movements in fractions of a degree, it remains to be seen if detecting changes at that magnitude results in clinically meaningful changes in function.

A lack of interim measures within stroke rehabilitation studies has been previously debated as a confounding issue that constrains dose-response investigations [72]. We believe that the inclusion of daily RMSE tracking data provides reliable, quantifiable measures of motor performance. Further, this data can be implemented on a trial-by-trial basis, offering preliminary insight into dose-response relationships that employ RMSE tracking data. Although the repeated measures analyses did not detect significant effects of time on RMSE, even a cursory look at the pre- and postintervention results indicates that moderately large differences exist at the end of treatment. With decreases in RMSE appearing within a few days of initiating the training and persisting until the end of treatment, these results give insight to future studies that may investigate further dose-responses beyond the 15 training days investigated in this study.

Although previous studies have demonstrated that additional therapy provides incremental benefits in behavioral outcomes [73], work by Dromerick et al. demonstrated that high dose therapy showed significantly less improvement at 90 days [74] when compared to low-dose therapy. This growing body of evidence presumably indicates an “inverted U” shaped curve, where too much or too little therapy results in worse outcomes. This discrepancy in the literature highlights the unmet need for rehabilitation tools to monitor dosing and performance to optimize the rehabilitation paradigms to hone in on the vertex of the “inverted U” curve.

Previous robotic-assisted therapy studies have identified limited benefit of RT at a low training time [7, 75, 76]. Therefore, this pilot study aimed to provide 30 hours of TDS-HM training to deliver an intervention dose that is consistent with other studies and has been previously shown to positively impact function and reduce impairment [7, 75]. Despite aiming for 30 hours of TDS-HM training, the total mean TDS-HM training time was 371.86 (range 53.58 to 687.83) minutes during the 5-week period. This is a 131% difference and far lower than that reported in most previous RT studies [6, 41, 67, 70, 77]. It can be argued that the TDS-HM training time might have been subtherapeutic. The heterogeneous dosing documented in the present study may also help to account for the magnitude of improvements observed in AROM. To assist with interpreting the findings, exploratory analysis of individual stroke survivor's AROM data revealed that four of the participants demonstrated improvements in AROM. This exploratory analysis suggested that the TDS-HM intervention might elicit the greatest improvements in AROM for stroke survivors with moderate deficits, a theme that is not apparent in pooled data.

Although low mean training time must be considered as a limitation of this pilot study, the trend was toward improved motor performance and decreased functional impairments. The trend shows promise, when considering that the confounding effects of spontaneous recovery might not have contributed to significant motor gains for the chronic stroke survivors, not active in other therapeutic interventions [78, 79]. It must also be considered that the findings from this study represent the presence of diminishing benefit after a maximally efficacious dose is reached [74]. Alternatively, given the low mean training time, it seems more likely that the stroke survivors did not reach the maximally efficacious dose prior to completion of the TDS-HM intervention. Determining the optimal dosing of intensity and timing of therapy after a stroke remains to be answered.

Another interpretation as to why several pre/postassessments did not reach significance may be that this preliminary study lacked sufficient power to detect any significant effects even if they exist, which is reasonable given the small sample size. Nonetheless, all measures show positive trends, with several measures displaying moderate to large differences between means, so utilizing the current result for estimations of effect size to power future studies appears to be justified. Future studies that involve the TDS-HM intervention and examine pre/posteffects for RMSE (Cohen's *d* = 2.957) require 4 participants per group for an alpha set a 0.05 and 80% power. Those that use as an outcome such as FMA/UE (Cohen's* d* = 0.331), PROM (Cohen's* d* = 0.892), AROM (Cohen's* d* = 0.367), SIS strength domain (Cohen's* d* = 0.486), and SIS hand domain (Cohen's* d* = 0.261) require 74, 12, 61, 36, and 118 subjects per group, respectively. These sample size estimates represent the minimum number of participants required to be sensitive to our calculated effect size.

While we made considerable effort to design a sound study, there are several limitations. First, although the target treatment time was initially set at 30 hours over 5 weeks, large heterogeneity in participant ability impaired participants from attaining the treatment dose of 30 hours. Second, a single group study design with multiple time points, instead of two-group randomized controlled trial, was used. Studies without a placebo or randomized comparison group may leave our results open to many possible interpretations and explanations. Thirdly, due to varied exposure and lack of randomization to the three-waveform trials, only the sine waveform was included in analyses, which limits the utility of waveform exposure beyond the initial sine wave. Finally, although a preliminary dose-response relationship might have been observed, the complex interaction of varied treatment dose over the 15-day intervention requires cautious interpretation.

Future studies involving the TDS-HM will address heterogeneous training volume by holding the training dose constant across the entire intervention. Monitoring cumulative training time and allowing the number of sessions to increase or decrease to accommodate the literature supported 30 hours recommended for UE rehabilitation [45], will ensure a dose match across participants and experimental groups. Randomization of waveform exposure will allow inclusion of all waveforms in future analyses.

Although it has been observed that longer daily treatment time is a significant predictor of motor performance improvement [5], investigating the potential for “inverted U” dose-response curve representing the presence of diminishing benefit after a maximally efficacious dose presents an interesting engineering solution for future studies utilizing RMSE tracking performance. Future TDS-HM studies will implement a novel software-based monitoring system that continuously provides a participants' retrospective RMSE for a given timeframe. Repeated, large deviations in RMSE may play a role in objectively determining the need for rest breaks. If the trend continues, task failure may have occurred showing the presence of a maximally efficacious dose of TDS-HM training.

Although robotic therapy is rarely implemented in severely impaired stroke survivors due to a lack of voluntary movement [80], future studies involving the TDS-HM will include a dose-matched HM control group with inert TDS. We hope this comparison will elucidate the additive benefits synchronous tongue and hand movements have for severely impaired stroke survivors. Additionally, future work will also explore the theoretical constructs of topographical reorganization in the tongue and hand motor cortexes through functional and structure neuroimaging techniques [81, 82].

## 5. Conclusion

The results of this pilot study are promising, demonstrating that the TDS-HM system may be a viable option for those who have survived a stroke with little to no voluntary UE movement. Data from this pilot study indicates that the TDS-HM can elicit clinically and statistically significant improvements, reducing UE impairments in moderately to severely impaired stroke survivors. These data are more notable when the relatively low total training time, compared to previous RT studies, is considered. This pilot study also provides preliminary insight into the volume of treatment time required to improve outcomes using this device. We also recognize the limitations of this preliminary study and are encouraged that the TDS-HM intervention provided haptic and visual feedback to encourage the stroke survivors to control the movements of their UE, a central component in stroke rehabilitation and initiating neural plasticity. The observed results offer important insights toward the potential use of hybridized robotic therapy and assistive technologies to tap into additive effects and the therapeutic implications in individuals who are unable to participate in stand-alone robotic therapy.

## Figures and Tables

**Table 1:** Baseline characteristics of the participants.

Baseline characteristics	Participants (*n* = 6)
Mean age at enrollment in years (SD)	60.8 (11.6)

Gender F and M *n* (%)	2 and 4 (66.67)

Mean time since stroke in months (SD)	21 (9.26)

Right hemiparesis (%)	3 (50)

Right hand dominant (%)	6 (100)

Incomplete data (%)	1 (16.67)

Baseline FMA/UE (SD)	25.67 (14.88)

Etiology of stroke	Ischemic (P1)
	Ischemic, received tPA and thrombectomy (P2)
	Hemorrhagic (P3)
	Ischemic (P4)
	Ischemic with hemorrhagic transformation (P5)
	Ischemic (P6)

Stroke location	Left subcortical MCA territory (P1)
	Right cortical MCA territory with subcortical sparing (P2)
	Right basal ganglia (P3)
	Left cortical MCA territory with subcortical sparing (P4)
	Left basal ganglia (P5),
	Right cortical MCA territory (P6)

P: participant; tPA: tissue plasminogen activator; MCA: middle cerebral artery; linear regression models exploring preliminary dose-response relationship between RMSE and daily sine wave practice time for all participants.

**Table 2:** Clinical Outcome Measures.

Outcome measures	Baseline (SD)	Postintervention (SD)	Mean difference from baseline (%)	95% CI	*p* value
RMSE	17.40 (3.89)	8.20 (2.06)	9.20 (52.87)	2.87 to 11.36	0.007^*∗*^
WMFT					
Total	5.29 (3.14)	5.13 (2.63)	−0.161 (3.02)	−1.72 to 1.39	0.801
Tasks incomplete in 120 s	5.71 (5.71)	5.43 (6.13)	0.28 (5.03)	−0.59 to 1.17	0.457
UE-Fugl-Meyer	25.67 (14.88)	31.17 (18.35)	5.5 (21.43)^†^	−0.00329 to 11.00	0.05^*∗*^
Range of motion					
Active	16.33 (22.36)	25.5 (27.58)	9.17 (56.15)	−1.99 to 20.32	0.088
Passive	53.50 (18.72)	69.83 (17.89)	16.33 (30.52)	1.81 to 30.85	0.034^*∗*^
Stroke Impact Scale					
Hand	30.00 (27.02)	36.67 (24.01)	6.67 (22.23)	−31.12 to 44.46	0.669
ADL	65.00 (29.37)	70.42 (28.13)	5.42 (8.34)	−8.99 to 19.83	0.378
Mobility	70.83 (35.08)	73.61 (31.17)	2.78 (3.92)	−4.82 to 10.38	0.39
Participants	43.23 (20.77)	47.40 (16.34)	4.16 (9.65)	−9.23 to 17.55	0.46
Strength	45.83 (26.42)	57.29 (20.70)	11.46 (25)^†^	1.80 to 21.11	0.028^*∗*^
Physical	57.89 (27.23)	63.54 (25.35)	5.65 (9.76)	−4.17 to 15.48	0.199

*Abbreviations*. RMSE: root mean squared error; WMFT: Wolf Motor Function Test; ^*∗*^*p* ≤ 0.05; ^†^achieved the previously validated MCID; linear regression models exploring preliminary dose-response relationship between RMSE and daily sine wave practice time for all participants.

**Table 3:** Total device usage in minutes for all waveforms training (SD).

	Mean	P1	P2	P3	P4	P5	P6
	Usage (min)						
Day 1	17.75 (7.54)	19.22	23.23	15.60	28.66	11.36	8.41
Day 2	26.48 (18.17)	16.78	40.70	39.06	47.80	8.75	5.80
Day 3	26.55 (22.65)	16.51	30.13	44.41	60.08	4.02	4.16
Day 4	31.17 (20.70)	15.53	33.00	50.01	59.38	24.32	4.77
Day 5	34.00 (23.47)	24.20	31.04	59.48	64.60	20.33	4.37
Day 6	25.15 (19.45)	18.89	30.17	61.02	20.97	15.41	4.45
Day 7	28.85 (24.63)	20.73	34.37	75.09	22.08	16.39	4.41
Day 8	22.42 (13.15)	21.02	28.54	42.56	24.95	13.16	4.32
Day 9	21.88 (12.48)	20.67	28.81	40.03	24.82	12.36	4.55
Day 10	23.31 (12.89)	20.77	29.59	44.18	24.78	12.22	8.34
Day 11	22.07 (14.91)	23.44	29.78	43.15	24.57	11.49	NA
Day 12	22.61 (14.99)	24.38	30.37	43.19	25.86	11.88	NA
Day 13	23.41 (15.42)	24.43	32.54	43.84	27.36	12.30	NA
Day 14	23.46 (15.24)	27.15	31.22	43.32	27.04	12.04	NA
Day 15	22.74 (15.05)	23.32	32.42	42.89	25.43	12.36	NA

P: participant; N/A: not applicable; linear regression models exploring preliminary dose-response relationship between RMSE and daily sine wave practice time for all participants.

## Abbreviations

HM: Hand Mentor
TDS: Tongue Drive System
TDS-HM: Hybrid Tongue Drive System and Hand Mentor intervention
UE: Upper extremity
RT: Robotic therapy
AROM: Active range of motion
PROM: Passive range of motion
ADL: Activities of daily living
RMSE: Root mean square error
FMA/UE: Upper extremity portion of the Fugl-Meyer Motor Assessment
WMFT: Wolf Motor Function Test
SIS: Stroke Impact Scale
AR(1): First order autoregression
MCID: Minimal clinically important difference
GUI: Graphical user interface
PCA: Principal component analysis
MCA: Middle cerebral artery
NIDDM: Non-insulin-dependent diabetes mellitus
ASA: Aspirin
tPA: Tissue plasminogen activator.

## Data Access

The datasets supporting the conclusions of this article are available in the FigShare repository at https://figshare.com/s/8d5a9a29057629a529d2 and https://figshare.com/s/d54f97b79dd0e05231bd.
